# Premature Ventricular Contractions and Non-sustained Ventricular Tachycardia: Association with Sudden Cardiac Death, Risk Stratification, and Management Strategies

**Published:** 2010-08-15

**Authors:** Seth H Sheldon, Joseph J Gard, Samuel J Asirvatham

**Affiliations:** 1 Department of Internal Medicine, Mayo Clinic, Rochester, Minnesota; 2 Division of Cardiovascular Diseases, Mayo Clinic, Rochester, Minnesota; 3 Department of Pediatrics and Adolescent Medicine, Mayo Clinic, Rochester, Minnesota

**Keywords:** PVC, premature ventricular contractions, ventricular tachycardia, ablation, sudden death

## Abstract

Premature ventricular contractions (PVCs) and non-sustained ventricular tachycardia (NSVT) are frequently encountered and a marker of electrocardiomyopathy. In some instances, they increase the risk for sustained ventricular tachycardia, ventricular fibrillation, and sudden cardiac death. While often associated with a primary cardiomyopathy, they have also been known to cause tachycardia-induced cardiomyopathy in patients without preceding structural heart disease. Medical therapy including beta-blockers and class III anti-arrhythmic agents can be effective while implantable cardiac defibrillators (ICD) are indicated in certain patients. Radiofrequency ablation (RFA) is the preferred, definitive treatment in those patients that improve with anti-arrhythmic therapy, have tachycardia-induced cardiomyopathy, or have certain subtypes of PVCs/NSVT. We present a review of PVCs and NSVT coupled with case presentations on RFA of fascicular ventricular tachycardia, left-ventricular outflow tract ventricular tachycardia, and Purkinje arrhythmia leading to polymorphic ventricular tachycardia.

## Introduction

Heart disease remains the leading cause of death in the United States [1].  Most of the 425,000 deaths per year attributable to heart disease are due to sudden cardiac death (SCD) [1,2]. Despite a reduction in the incidence of SCD, primary prevention remains challenging, as SCD is the initial presentation of cardiac disease in many patients. Subtle clues to the presence of electrocardiomyopathy in these patients may have been absent or missed.

The presence of premature ventricular contractions (PVCs) and non-sustained ventricular tachycardia (NSVT) can be a marker of electrocardiomyopathy. While often considered benign, these types of ventricular ectopy can have negative consequences through an increased risk of SCD, underlying cardiomyopathy, or induction of cardiomyopathy. We discuss the relationship of PVCs and NSVT to SCD and cardiomyopathy, risk stratification strategies, and approaches to management including common scenarios for ablation.

## Sudden Cardiac Death

The estimated incidence of SCD in the United States is 300,000 deaths annually [2]. Commonly recognized risk factors include ischemic heart disease, previous cardiac arrest, and cardiomyopathy. Nearly 25% percent of deaths, however, occur in the general adult population with previously silent or unrecognized cardiac disease.  As the overall incidence of SCD in the Unites States is 0.1-0.2% per year, it would be challenging to apply a population-wide screening test or intervention. Much of the focus has been upon prevention and identification of ischemic heart disease, as this accounts for approximately 80% of SCD [2].

The overall incidence of SCD appears to be decreasing [3]. Evidence in patients with out-of-hospital cardiac arrest suggests that this reduction may be limited to patients with ischemic cardiac disease. The incidence of ventricular fibrillation outside the hospital arrest in patients from Rochester, MN with ischemic heart disease decreased while those with non-ischemic heart disease increased from 1991-2004 [4]. The gains may be attributable to improved primary and secondary prevention of coronary artery disease as well as advanced management strategies, including early reperfusion. Recent implementation of automated external defibrillators would be expected to reduce the incidence of SCD further in both groups. The use of therapeutic hypothermia improves outcomes for SCD in patients who have persistent coma after return of spontaneous circulation [4,5].

Since our currently identified risk factors fail to account for much of the population at risk for SCD and the incidence of SCD may be increasing in patients without ischemic heart disease, we must refocus our attention on this group, thus the need to identify new risk factors to capture a larger segment of those at risk.  Furthermore, we must recognize clues suggesting subtle electrocardiomyopathy.

## Definitions and Epidemiology

**Figure 1:**
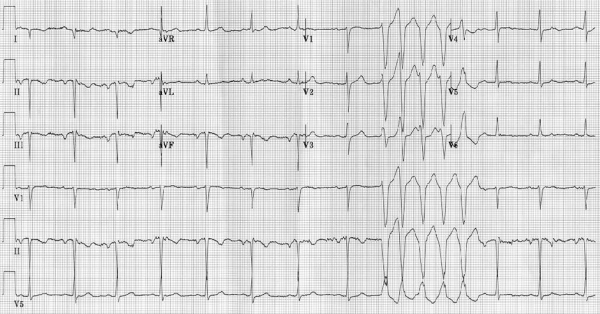
Electrocardiogram demonstrating sinus rhythm with a five beat run of nonsustained ventricular tachycardia with left bundle branch block morphology (negative in V1).

PVCs are early depolarizations originating in the ventricle due to increased automaticity. NSVT occurs when three or more consecutive PVCs occur at a rate greater than 100 beats-per-minute (Figure 1). They may be monomorphic or polymorphic and are often present in patients presenting with nonspecific cardiac symptoms. While PVCs and NSVT are frequently seen in the general population and are sometimes considered clinically insignificant, they mark a population at increased risk for cardiac disease including SCD and cardiomyopathy.

The incidence of PVCs and NSVT in the adult population varies. Ambulatory 24-hour ECG monitoring in male medical students identified PVCs in 50% of the cohort, but only 2% had more than 50 PVCs in 24 hours [6]. A Framingham Offspring Study in patients without known cardiac disease reported PVCs during exercise testing in 27% of patients. Those with PVCs had a statistically significant but small increased mortality hazards ratio of 1.71-1.86 [7]. Patients with heart disease have a higher incidence of complex or frequent arrhythmia [8]. Overall, ventricular ectopy appears to be a frequent finding with a small but statistically significant increased sudden cardiac death and mortality risk [9].

## Presentation and Pathophysiology

The presentation of PVCs and NSVT varies and may include palpitations, chest pain, pre-syncope, syncope, or heart failure. Dysrhythmias occur with increased automaticity, reentry, or triggered automaticity.  PVCs arise from tissue with increased automaticity. This is influenced by autonomic tone, ischemia/reperfusion, systemic abnormalities such as electrolyte disturbances, and cardiotoxic factors [2]. Monomorphic VT typically occurs with reentry around a substrate or due to triggered automaticity as seen with right-ventricular outflow-tract tachycardia. Polymorphic VT is typically the result of increased automaticity.

There are three major concerns regarding the presence of PVCs and NSVT. The substrate often associated with monomorphic VT increases the risk for sustained VT. Another concern, particularly with increased automaticity and polymorphic VT, is that a ventricular beat may be coupled closely with the preceding QRS complex and produce ventricular fibrillation. In addition, frequent ventricular ectopy is known to adversely affect cardiac function itself, namely tachycardia-induced cardiomyopathy.

## Relationship to Cardiomyopathy: Primary arrhythmia or primary cardiomyopathy

**Figure 2:**
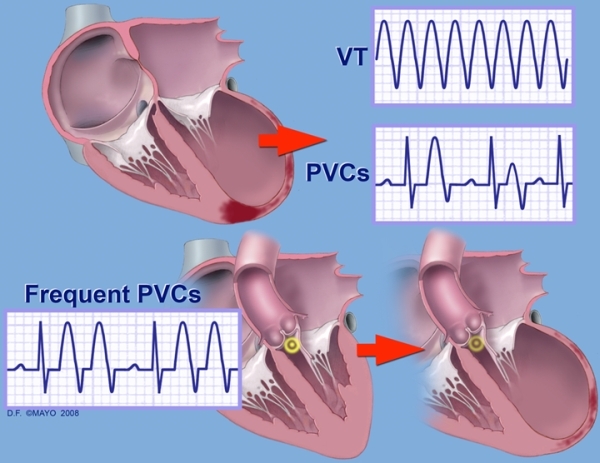
The upper portion of this figure depicts ectopy that can occur after myocardial injury has caused ventricular dysfunction.  Frequent PVC's may also cause ventricular remodeling as seen in the lower portion of the figure. VT = ventricular tachycardia; PVCs = premature ventricular contractions

It can be difficult to determine whether a primary cardiomyopathy is present resulting in ventricular ectopy or a primary arrhythmia causing tachycardia-induced cardiomyopathy (Figure 2) [9]. While supraventricular arrhythmias and ventricular tachycardias are recognized mediators of tachycardia-induced cardiomyopathy, PVCs have recently been shown to cause tachycardia-induced cardiomyopathy [10,11]. Differentiating a primary cardiomyopathy from tachycardia-induced cardiomyopathy is essential, as the latter may be reversible, particularly by radiofrequency ablation [12].

**Figure 3:**
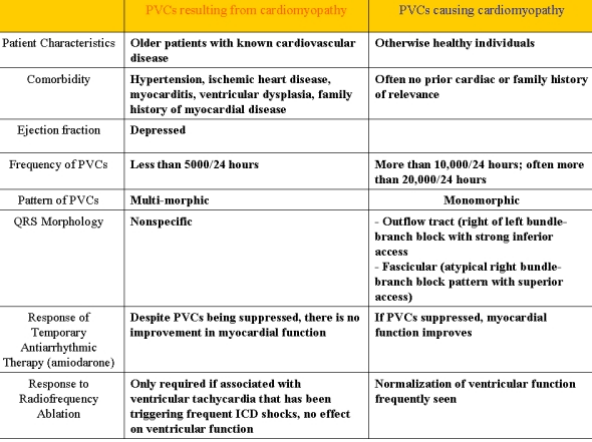
Characteristics of primary cardiomyopathy versus tachycardia-induced cardiomyopathy

Certain factors are helpful in determining the primary disorder (Figure 3) [9]. Clues suggestive of a primary arrhythmia include 1) young, healthy patients without underlying cardiac disease; 2) frequent ventricular ectopy (often >20,000 beats per day); and 3) one to two primary ectopy morphologies. Ultimately, improvement in ventricular function with suppression of the arrhythmia via antiarrhythmic therapy or radiofrequency ablation (RFA) defines tachycardia-induced cardiomyopathy.

## Evaluation and Risk Stratification

An evaluation of patients with PVCs and NSVT should determine the extent of symptoms, evidence of underlying cardiomyopathy, the risk for tachycardia-induced cardiomyopathy, and the risk for sudden cardiac death. This begins with a focused history including associated symptoms, frequency, duration, triggers, family history, and risk factors for coronary artery disease. The physical exam should evaluate for evidence of systemic disease (such as connective tissue diseases, thyroid abnormalities, sarcoidosis, or amyloidosis).

Routine tests include a resting ECG, 24-hour ambulatory electrocardiography, and an echocardiogram. The ambulatory electrocardiogram can establish the frequency, duration, and morphology of ectopy. Early repolarization on electrocardiogram is prognostic of increased risk of death from cardiac causes [12]. The echocardiogram provides the left ventricular function and helps in the evaluation for underlying cardiomyopathy. Patients at risk for or with suspected coronary heart disease may require stress testing or coronary angiography. Additional diagnostic evaluation such as cardiac magnetic resonance imaging may be warranted depending on individual patient factors.

Noninvasive and invasive methods of risk stratification for SCD are available. The most recognized risk factor is diminished left ventricular function as determined by the ejection fraction. This, however, fails to account for the majority of SCD cases [13]. Evaluation for underlying coronary heart disease can be helpful as this accounts for approximately 80% of SCD, although it has a low predictive power and is often non-specific [14]. Additional noninvasive methods are available including ECG markers, assessment of baroreceptor sensitivity, and measurement of heart-rate variability. The ECG and SAECG may be used to evaluate QRS duration, for the presence of T-wave alternans, and for QT prolongation [13]. The presence of differing types of PVCs or NSVT increases the risk of polymorphic ventricular tachycardia or VF. The primary invasive method for risk stratification remains an electrophysiology study. Using the gathered data, we must determine which patients to offer medical therapy, implantable cardiac defibrillators (ICD), or RFA.

## Therapy: Implantable cardiac defibrillators

ICDs are recognized as the principal method of preventing sudden cardiac death in the highest-risk population. The AVID study in patients with history of sudden cardiac arrest (secondary prevention) showed a reduction of overall mortality and SCD incidence with ICDs as compared with amiodarone [15]. MADIT II evaluated patients with left ventricular ejection fractions of less than 30% with history of previous myocardial infarction and showed a reduction in overall mortality with ICDs versus no ICD [16]. SCD-HeFT demonstrated the efficacy of ICDs in both ischemic and non-ischemic cardiomyopathy [17]. Unfortunately, the groups known to benefit from ICD therapy comprise a minority of the overall population at risk for sudden cardiac death.

PVCs and NSVT increase the risk for sudden cardiac death. There is, however, little evidence evaluating the efficacy of ICDs in this population. The MADIT I study showed a survival benefit with ICD therapy versus conventional therapy in patients with asymptomatic NSVT and symptomatic ischemic cardiomyopathy with an ejection fraction of less than 35% [18]. The DEFINITE study in patients with non-ischemic dilated cardiomyopathy, ejection fraction <36%, and NSVT or greater than 10 PVCs per hour demonstrated a reduction in overall mortality and sudden cardiac death with ICD therapy and conventional therapy versus conventional therapy alone [19]. Although PVCs or NSVT are components of the inclusion criteria in these studies, the study populations were limited to those with a diminished ejection fraction and thus were not inclusive of the major population at risk for SCD.

Economic constraints and quality of life implications must be entertained when considering ICD therapy. A reduction in the incidence of sudden cardiac death may not equate to survival with meaningful quality of life [20]. Furthermore, the absolute risk reduction of SCD in the majority of patients with PVCs or NSVT may not warrant the cost of a $17,500 device [21]. In the absence of evidence to guide the use of ICDs in patients with PVCs or NSVT without a diminished ejection fraction, routine use of ICD therapy is not recommended. Further evaluation will be needed to determine the patients in this risk group that would benefit from ICD therapy. Presently, we must decide based on clinical presentation and electrocardiographic data which patients to offer medical therapy versus radiofrequency ablation.

## Therapy: Medical

The goal of therapy for PVCs and NSVT is to 1) alleviate symptoms; 2) suppress ventricular tachyarrhythmias and PVCs; 3) prevent or reverse tachycardia-induced cardiomyopathy; and 4) decrease shocks in patients with ICDs. Lifestyle modifications such as reducing caffeine and alcohol intake can be recommended initially, although neither moderate use nor reduction in intake has a major effect on ventricular ectopy [16,17].

Beta-blockers can be considered for treatment of ventricular tachyarrhythmias. They decrease mortality in patients post-myocardial infarction [18] and in heart failure [19], at least partially due to a reduction in the incidence of SCD. Beta-blockers also reduce mortality in patients with ischemic cardiomyopathy with EF ≤ 40% and NSVT [22]. There is evidence showing a reduction in PVCs in the absence of a reduced EF [20,23]. In the setting of ventricular arrhythmia originating in the right ventricular outflow tract (RVOT), atenolol has been shown to modestly reduce PVCs with no statistically significant reduction in symptoms compared with placebo  [21]. Beta-blockers are largely well-tolerated and, given a survival benefit in patients with ventricular arrhythmias, are a reasonable first-line drug but unlikely to provide definitive therapy.

The role of antiarrhythmic drugs in the management of PVCs and NSVT is best assessed by evaluating their efficacies versus side-effect profiles, including the propensity for pro-arrhythmic effects. Selection of antiarrhythmic drugs may be empiric, guided by the use of electrophysiology study with stimulation testing, or guided by ambulatory/exercise ECG monitoring [24].

Arrhythmia suppression with class I antiarrhythmic drugs is not advised due to the results of the CAST trial [25]. Although effective in suppressing PVCs, the use of encainide and flecainide was associated with increased mortality in patients with history of myocardial infarction. This was attributed to proarrhythmic effects in the drug class, especially in patients with left-ventricular dysfunction. Thus, class I antiarrhythmic agents are generally not recommended for suppression of PVCs or NSVT in patients with coronary disease.

Class III antiarrhythmic drugs have been evaluated in patients at risk for SCD. The Amiodarone Trials Meta-Analysis Investigators showed a 13% reduction in mortality and 29% reduction in SCD in patients with recent myocardial infarction or congestive heart failure treated with amiodarone versus placebo [26]. The SCD-HeFT trial, meanwhile, did not show a reduction in mortality with amiodarone in patients with New York Heart Association Class II or III heart failure with a left ventricular ejection fraction of less than 36% [17]. The discrepancy between these studies might be due to a higher mean LVEF in the former study.

Another class III drug, dofetilide, has been studied in the setting of inducible sustained ventricular tachycardia. It appears to be as efficacious as sotalol and better tolerated, possibly due to less beta-blockade [27]. Dofetilide has a reasonable safety profile but little is known about the efficacy in PVCs and NSVT [28,29].

Given the pro-arrhythmic effects and adverse side effect profiles of most antiarrhythmic drugs, it is often impractical to use them for primary prevention of SCD. Nevertheless, in patients with structural heart disease and symptomatic or frequent ventricular ectopy, amiodarone or dofetilide could be used with caution. They may also be utilized short-term in the setting of suspected tachycardia-induced cardiomyopathy to evaluate for improvement of ejection fraction that might be sustained with radiofrequency ablation.

## Treatment: Radiofrequency ablation

RFA is an additional management option for PVCs and NSVT.  Determining the target site for ablation depends on the mechanism of the arrhythmia. Activation mapping is used with PVCs to identify the earliest activation point, representing the automatic tissue targeted for ablation. Alternatively, pace mapping can be utilized to find the paced site that matches spontaneous premature ventricular depolarization [22]. Common targets for RFA in primary arrhythmia disorders include: the right ventricular outflow tract [23] left ventricular outflow tract [30,31], papillary muscle [32] and the fascicles [33-35].

RFA has been successful in alleviating symptomatic ventricular ectopy [23], reducing ICD shocks [36], and reversing tachycardia-induced cardiomyopathy [36]. There are, nevertheless, several associated risks that vary depending on the site of ablation and amount of energy delivered. These include bleeding, infection, cardiac perforation, coronary artery injury, and thromboembolism. Technological advances such as advanced electroanatomic mapping and intracardiac ultrasound [37] have made RFA safer and thus a viable option in certain patients with PVCs and NSVT. Further study will be needed to determine the long-term efficacy of RFA for ventricular ectopy.

## Management algorithm

**Figure 4:**
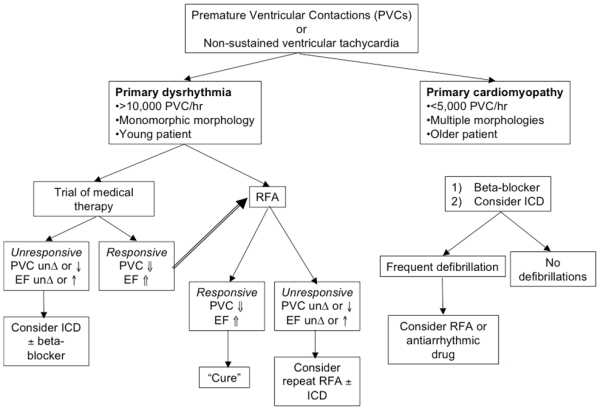
Management algorithm for PVCs or NSVT.

Figure 4 is a proposed management algorithm. Determining the primary disorder is essential as arrhythmia suppression may reverse tachycardia-induced cardiomyopathy. Patients with a primary arrhythmia responding to medical therapy should be considered for RFA. It is also important to consider the patient's risk for SCD and the role for ICD.

## Case 1: 19 year-old female with dyspnea on exertion

**Figure 5:**
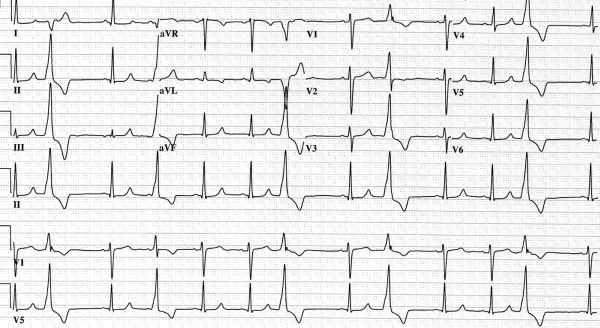
Electrocardiogram demonstrating underlying sinus rhythm with frequent monomorphic PVCs.  The morphology of the PVCs suggests a LVOT focus. The first clue is the right bundle branch block morphology of the PVCs suggests left-sided origin. The second clue is that the PVCs are strongly positive in inferior leads (II, III, aVF) and strongly negative in aVR and aVL which suggests the origin is superior in the outflow tracts.

Monomorphic ectopic complexes strongly suggest a primary arrhythmic propensity as seen in the following illustrative case. A 19 year-old female presented with fatigue and dyspnea on exertion. Her electrocardiogram (Figure 5) demonstrated sinus rhythm with monomorphic PVCs. A Holter monitor recorded 19,000 PVCs in 24 hours.

She had a reduced left ventricular ejection fraction (32%) without evidence of obstructive coronary disease or anomalous anatomy. High dose beta-blocker therapy was poorly tolerated and ineffective at controlling her PVCs. Similarly, sotalol 120 mg by mouth twice daily caused fatigue and only minimal reduction in her PVCs.

**Figure 6:**
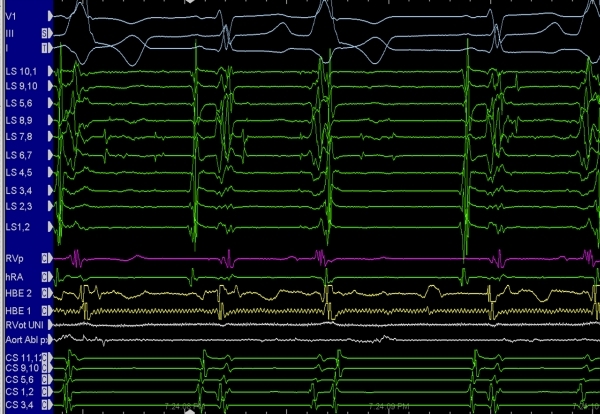
Intracardiac electrograms and EKG in a patient with an unusual supravalvar origin for PVCs.  EKG shows PVCs occurring in a begiminal pattern.  LS is a circumferential multielectrode catheter placed in the aortic route.  Note: small, spike-like signals that occur with or without related exit to the ventricle producing PVCs.  RVT = right ventricular; HRA = high right atrium; HBE1 and HBE2 = His bundle recording catheters; RVOT uni = right ventricular outflow tract unipola; AORT ablation = ablation catheter placed in the distal aortic outflow tract; CS = coronary sinus.

Since medical therapy was not effective for her, she was referred for an EP study. The morphology of her PVCs suggested an outflow tract origin given they were strongly positive in the inferior leads (II, III, aVF) and strongly negative in aVL and aVR.  The right bundle branch morphology (positive in V1) and S wave in lead I suggested a left-sided focus such as from the left ventricular outflow tract (LVOT) [38]. During the EP study, a lasso catheter was placed in the LVOT where the focus was localized based on activation at the Sinus of Valsalva (Figure 6). She had symptomatic relief and resolution of her PVCs after ablation.

The outflow tracts are common sources of PVCs and have classic ECG patterns. The key electrocardiographic findings for an LVOT focus are illustrated above. An RVOT focus similarly has strongly inferior vector (positive II, III, aVF, negative in aVR and aVL) but with a left bundle branch block pattern (positive in V1). Arrhymothogenic right ventricular cardiomyopathy [39] is important to recognize when working up patients with outflow tract arrhythmias and appropriate imaging should be pursued.

## Case 2: 17 year-old hockey player with pre-syncope and palpitations

**Figure 7:**
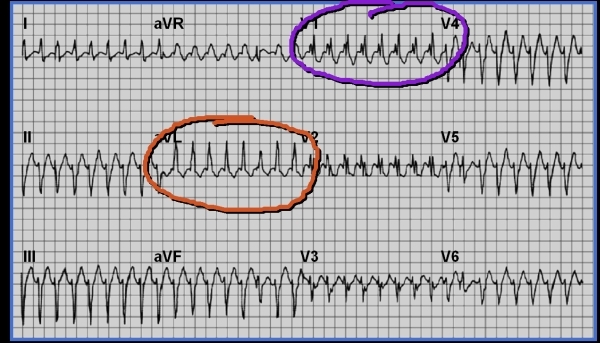
Typical electrocardiographic pattern of idiopathic left ventricular tachycardia (fascicular VT).  A typical right bundle morphology is noted in lead V1. Unlike outflow tract VT, however, leads AVl and AVr are both positive. A strong superior axis is noted, and the positive R wave dominates in lead one.

**Figure 8:**
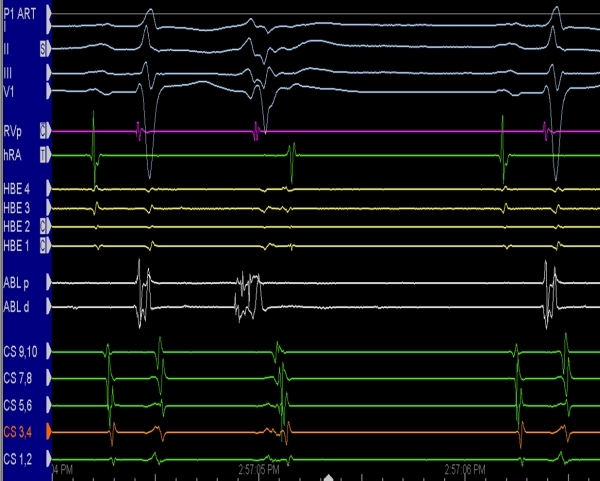
Intracardiac tracings obtained when mapping a constant PVC that initiated ventricular fibrillation. Note the spike-like signals on the ablation electrodes (ABLD = ablation distal; ABLP = ablation proximal). In this instance, these signals represented the depolarization of the arrhythmogenic Purkinje fibers responsible for the arrhythmia. HBE = His bundle; RV = right ventricle; HRA = high right atrium; CS = coronary sinus

Fascicular ventricular tachycardia [33] is a specific arrhythmia characterized by a relatively narrow QRS complex with a left bundle-branch block pattern that occurs in structurally normal hearts. It may predispose to arrhythmic events and possibly tachycardia induced cardiomyopathies. A 17 year-old hockey player presented with palpitations, presyncope and an abnormal electrocardiogram (Figure 7) demonstrating fascicular ventricular tachycardia that was confirmed by EP study (Figure 8) and was treated by ablation.

These two cases examples demonstrate that primary arrhythmias may develop in patients who do not have a traditional cardiomyopathy. The astute clinician is mindful of the uncommon cardiomyopathies manifesting rhythm abnormalities.

## Case 3: 31 year-old with polymorphic VT

**Figure 9:**
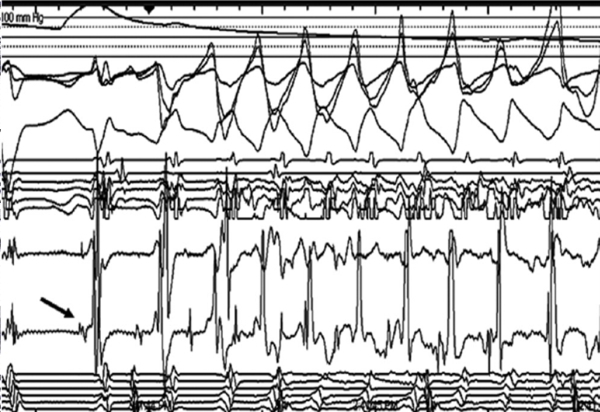
EP tracing demonstrating the onset of ventricular fibrillation provoked by a PVC.  The arrow points to a Purkinje potential that precedes the PVC.  Note that the time interval from the Purkinje potential to the onset of the QRS varies likely because of different exit sites)

Polymorphic ventricular tachycardia can, in specific circumstances, be treated by ablation. A 31 year-old female collapsed after exercising and had a second episode while shopping. Polymorphic ventricular tachycardia / ventricular fibrillation was diagnosed, and her electrocardiogram had a borderline QT interval. She had no structural heart disease by echocardiogram and she did not provoke any evidence of arrhythmia during an exercise test. An ambulatory Holter monitor demonstrated repeated runs of minimally-symptomatic polymorphic VT with a normal QT interval. An ICD was implanted and subsequent interrogation demonstrated similar episodes with frequent detections and shocks. EP study found Purkinje potentials preceding PVCs (Figure 9) that induced polymorphic VT.  Using pacemapping, the fascicular focus was identified and ablated [32].

## Conclusion

PVCs and NSVT are frequently encountered in clinical practice. Their presence may increase the risk for more malignant dysrhythmias such as sustained VT or ventricular fibrillation that can cause sudden cardiac death. PVCs and NSVT can be associated with a reversible tachycardia-induced cardiomyopathy or a primary cardiomyopathy. A focused evaluation and implementation of risk stratification strategies are helpful in treatment selection. RFA is effective in alleviating symptoms and reducing ICD shocks, especially in the setting of outflow tract or fascicular tachycardia. The presented cases highlight key points in the approach to RFA for PVCs and NSVT.
